# Impact of fasting and refeeding on immune markers, hepatic gene expression, and gut microbiota in geese: insights into metabolic regulation and gut-liver interactions

**DOI:** 10.3389/fmicb.2025.1498460

**Published:** 2025-02-18

**Authors:** Yi Liu, Guangquan Li, Xianze Wang, Huiyan Jia, Jiuli Dai, Shufang Chen, Daqian He

**Affiliations:** ^1^Institute of Animal Husbandry and Veterinary Science, Shanghai Academy of Agricultural Sciences, Shanghai, China; ^2^Institute of Livestock and Poultry Research, Ningbo Academy of Agricultural Sciences, Ningbo, China

**Keywords:** fasting and refeeding, hepatic gene expression, gut microbiota, gut-liver interactions, immune markers, geese

## Abstract

Fasting and refeeding protocols, which induce short-term fluctuations in nutrient and energy levels, elicit adaptive physiological responses in animals. In this study, biochemical, transcriptome and 16S rRNA sequencing techniques were used to investigate the physiological effects of fasting and refeeding on immune responses, liver gene expression, and gut microbiota composition in geese. Fasting led to a significant reduction in circulating levels of IgA and IFN-*γ*, while IgG, TNF-*α*, IL-6, and IL-10 levels remained stable. Upon refeeding, IgA and IFN-γlevels rapidly returned to baseline. RNA-Seq analysis identified 858 differentially expressed genes (DEGs) between the control and fasted groups, and 732 DEGs between the fasted and refed groups. Key regulatory genes involved in energy metabolism and lipid biosynthesis, such as *CPT1A*, *HMGCS1*, and PCK1, were upregulated during fasting, reflecting an increase in fatty acid oxidation and gluconeogenesis. Conversely, lipogenic genes, including *FASN*, *ACSS2*, *ACCα*, and *SCD*, were downregulated during fasting and upregulated during refeeding, indicating a metabolic shift from catabolic to anabolic processes. Gene Ontology (GO) and KEGG pathway enrichment analyses revealed significant involvement of the PPAR signaling, glycolysis/gluconeogenesis, and insulin signaling pathways. Additionally, 16S rRNA gene sequencing indicated that fasting increased the abundance of Bacteroidetes and Proteobacteria, while decreasing Firmicutes. Both alpha and beta diversity were significantly reduced during fasting. Functional analysis of the gut microbiota suggested a shift toward fatty acid oxidation during fasting. Correlation analysis further demonstrated that the relative abundance of *Barnesiella* was positively correlated with genes involved in gluconeogenesis and negatively correlated with lipid metabolism genes, such as *ELOVL6* and *PHGDH*. This underscores the role of the gut-liver axis in regulating metabolic adaptations. These findings offer critical insights into how short-term fluctuations in nutrient availability influence immune function, metabolic regulation, and gut microbiota composition in geese. This research also provides potential strategies for optimizing poultry nutrition and health management.

## 1 Introduction

Feed is crucial in poultry farming, providing the essential nutrients and energy needed for both growth and reproduction. In recent years, poultry producers have focused on improving feed management strategies to optimize cost-efficiency. These strategies aim to enhance feed conversion rates, reduce fat content, and increase meat production, all contributing to greater overall farm productivity. Numerous studies have demonstrated that implementing early, appropriate restrictive feeding in broilers can improve feed efficiency, enhance carcass quality, reduce fat content, and lower the incidence of related health issues—all without negatively impacting growth. This, in turn, significantly reduces farming costs (Santoso, 2001; Nielsen et al., 2003; Pinheiro et al., 2004; Novel et al., 2009; Mohammadalipour et al., 2016). Similarly, early feed restrictions in ducks have been shown to achieve target weights and breast mass comparable to control groups while also reducing leg abnormalities (Van Blois et al., 2019; Bentley et al., 2020). Excessive feeding restrictions, however, can have detrimental effects on growth, bone development, intestinal microbiota balance, and gut permeability, ultimately increasing the risks associated with livestock farming (Bentley et al., 2020; Englmaierová et al., 2021; Li et al., 2021). Despite these findings, the impact of restrictive feeding on the growth and physiology of geese remains unexplored. Fasting and refeeding protocols, which cause short-term fluctuations in nutrient and energy levels, can mimic adaptive physiological responses in animals that are triggered by food restriction followed by refeeding. These systems are widely used to study the effects of dietary restrictions on various physiological functions, including immune responses, lipid metabolism, and disease progression (Nagai et al., 2019; Huang et al., 2020; Carulla et al., 2023; Zhong et al., 2023). In the realm of animal nutrition and physiology, the intestines and liver are not only crucial for the digestion, absorption, and metabolism of essential nutrients, but they also play a key role in regulating overall energy balance and maintaining the health of the organism. Therefore, this study utilized a fasting/refeeding protocol to examine the effects of short-term food availability fluctuations on the intestines and liver in geese.

The term “gut-liver axis” refers to the intricate functional and anatomical bidirectional relationship between the liver and the gut (Arab et al., 2018). This connection facilitates a continuous exchange between the intestine and liver through the portal vein and systemic circulation. Numerous studies have demonstrated that short-term dietary interventions can significantly affect both the intestinal microflora and liver metabolism (David et al., 2013; Wang et al., 2020; Chen et al., 2021). In contrast to most mammals, the liver in poultry primarily serves as the main site for the endogenous synthesis of fatty acids and lipoproteins (Hermier, 1997). The liver of geese, in particular, is highly sensitive and responsive to dietary changes. When geese are overfed with a high-energy, carbohydrate-rich diet, it leads to an imbalance in lipid synthesis, transport, and *β*-oxidation, causing rapid fat accumulation (Wei et al., 2021). Additionally, overfeeding influences intestinal physiology, alters the microbial community, and modifies the metabolic profile of the gut contents (Wei et al., 2020; Zhao et al., 2020). Previous research indicates that, under conditions of nutritional excess, the reciprocal interactions between the intestine and liver play a crucial role in regulating glycolipid metabolism, oxidative stress, and inflammatory responses (Wen et al., 2022; Lu et al., 2021). Dietary restriction exerts a significant influence on the metabolism of the avian liver, particularly affecting genes involved in energy metabolism, with a notable impact on glycolipid metabolism (Lindholm et al., 2022). Studies further demonstrate that fasting activates the adenosine monophosphate-activated protein kinase (AMPK) pathway in broiler chicken livers, leading to decreased expression of fatty acid synthase (*FAS*) and sterol regulatory element-binding proteins (*SREBP-1*), thereby reducing hepatic lipid synthesis during periods of energy deficiency (Wang et al., 2020). Additionally, fasting and refeeding profoundly alter liver metabolism, inducing a shift from lipolysis during fasting to enhanced lipogenesis upon refeeding (Cogburn et al., 2020). Research on geese livers suggests that fasting and refeeding regulate lipid metabolism largely via the peroxisome proliferator-activated receptor (PPAR) signaling pathway (Chen et al., 2021). Fasting promotes fatty acid oxidation while suppressing fatty acid synthesis, whereas refeeding reverses these effects. Although short-term fasting has been shown to increase intestinal permeability in chickens and influence the expression of intestinal structural proteins, lipid transport proteins, general stress response proteins, and intestinal defense proteins (Gilani et al., 2017; Simon et al., 2019), there remains a lack of comprehensive research on the effects of fasting on the avian gut microbiome. In conclusion, nutritional states, including overfeeding and dietary restriction, play a crucial role in regulating avian liver metabolism and the intestinal microbiome, with interactions along the gut-liver axis significantly affecting nutrient digestion, absorption, and metabolism. Specifically, overfeeding with a carbohydrate-rich diet leads to excess energy, which significantly enhances hepatic lipogenesis in geese, resulting in the development of fatty liver. This condition, known as fatty liver, is a key component in the production of foie gras, a specialty food product. These findings offer valuable insights into optimizing liver health and production efficiency in geese, which may have significant implications for improving foie gras production systems.

The Zhedong White goose is a distinguished medium-sized white goose breed in China, notable for its pristine white plumage, rapid early growth, and exceptional meat quality. This breed enjoys widespread popularity across China, with annual rearing numbers surpassing 30 million. Thus, we sought to investigate the effects of fasting and refeeding on various physiological parameters in Zhedong White geese, including serum immune and inflammatory markers, liver transcriptional profiles, and intestinal microbiota. Additionally, we examined the interactions within the gut-liver axis throughout this process. Our findings offer valuable insights into the physiological responses of geese to food restriction, contributing to a deeper understanding of the regulatory genes involved in hepatic glycolipid metabolism and their associated interaction networks, as well as their connections with intestinal microorganisms. Although research on the impact of food restriction on the gut-liver axis in geese remains limited, our study highlights the potential for optimizing feeding management strategies through a better understanding of this relationship.

## 2 Materials and methods

### 2.1 Ethics statement

The study was conducted in strict accordance with the 2017 Regulations of the State Council of the People’s Republic of China concerning the Management of Experimental Animals. It received formal approval from the Animal Ethics Committee of the Shanghai Academy of Agricultural Sciences (Shanghai, China) under approval number SAASPZ0522046. All procedures adhered to the ethical guidelines for conducting animal research.

### 2.2 Experimental animals and sample collection

The experiment was conducted at the Zhuanghang Research Farm, affiliated with the Shanghai Academy of Agricultural Sciences in Shanghai, China. A total of 108 one-day-old male Zhedong White Geese, all of similar weight, were selected for the study. These geese were incubated in a single batch at the research farm and randomly divided into three groups. Each group consisted of six replicates, with six geese per replicate. The geese were housed indoors on net beds measuring 6 m^2^ (3 × 2 m), maintaining a stocking density of 1 m^2^ per goose. The net beds were constructed from specially designed PVC manure leakage boards with 12 mm × 12 mm holes and were elevated 60 cm above the ground. Each pen was equipped with a plastic feeder, 30 cm in height and 35 cm in base diameter, and four nipple drinkers. Both the feeder and drinkers were adjustable in height to accommodate the growth stages of the geese. Standardized conditions were maintained throughout the study, including uniform room settings, a consistent supply of feed and water, and ad libitum access to food and water. During the first 14 days, the geese were exposed to continuous lighting (24 h) with an intensity of 4 to 5 W/m^2^. The room temperature was maintained between 28 and 32°C, with a relative humidity of 55–65%. From days 15 to 42, a 12-h light/dark cycle was implemented, with the same light intensity. The temperature was adjusted to 23–25°C and humidity to 50–60%. The feed provided followed the NRC (1994) standards, specifically formulated to meet the geese’s nutritional requirements at various developmental stages (Table 1). At 42 days of age, the first group continued on their regular feeding regimen, while the other two groups were subjected to fasting and refeeding protocols. The second group began fasting at 11:00 AM, and the third group at 8:00 AM, with free access to water during the fasting period. After a 24-h fast, the third group underwent a 3-h refeeding phase. At 43 days (11:00 AM), one representative sample from each replicate was selected, resulting in a total of 18 samples with an average weight of 1898.52 ± 84.22 g (six samples per group). Blood samples (5 mL) were drawn from the wing vein and immediately stored at 4°C for 20 min. The samples were then centrifuged at 3500 g for 15 min to separate the serum, which was subsequently stored at −20°C for the analysis of immune and inflammatory markers. Euthanasia was carried out using carbon dioxide inhalation followed by cervical dislocation, performed by trained personnel. Liver tissue samples, measuring 2 cm in length and 1.5 cm in width, were collected from the left lobe, approximately 2 cm from the liver’s edge. One portion of each liver sample was fixed in 4% paraformaldehyde at room temperature for histomorphological analysis, while the other portion was rapidly frozen in liquid nitrogen and stored at −80°C in 2 mL cryotubes for transcriptomic and gene expression studies. Additionally, rectal contents were collected from the section located 2 cm from the junction of the cecum and rectum, quickly frozen in liquid nitrogen, and stored at −80°C for future gut microbiota analysis.

**Table 1:** Feed ingredients and analyzed chemical composition of geese diets (air-dry basis %).

Ingredients	Content %	
	1–28 d	28–44 d
Corn	60.30	58.80
Soybean meal	32.60	25.60
Fish meal	2.00	10.10
Soybean oil	2.00	1.50
Lys + Met	0.10	0.00
Limestone	0.00	1.00
Premix^a^	3.00	3.00
Total	100	100
Nutritional level		
ME/(MJ/kg)	12.13	12.55
Crude protein	20.23	16.00
Crude fiber	3.07	7.00
Ca	0.55	0.68
P	0.45	0.43

^aOne kilogram of the premix contained the following: Fe 100 mg, Cu 8 mg, Mn 120 mg, Zn 100 mg, Se 0.4 mg, Co 1.0 mg, I 0.4 mg, VA 8330 IU, VB1 2.0 mg, VB2 2.8 mg, VB6 1.2 mg, VB12 0.03 mg, VD3 1,440 IU, VE 30 IU, biotin 0.2 mg, folic acid 2.0 mg, pantothenic acid 20 mg, and niacin acid 40 mg.^

### 2.3 Serum immune and inflammatory markers analysis

Serum immune and inflammatory markers, including Immunoglobulin A (IgA), Immunoglobulin G (IgG), Tumor Necrosis Factor-alpha (TNF-*α*), Interferon-gamma (IFN-*γ*), Interleukin-6 (IL-6), and Interleukin-10 (IL-10), were quantified during both fasting and refeeding periods. These measurements were performed using enzyme-linked immunosorbent assay (ELISA) kits from Shanghai Renjie Bio-technology Co., Ltd. (Shanghai, China), following the manufacturer’s specific protocols designed for goose samples. Optical density (OD) readings were taken at a wavelength of 450 nm using a multifunctional enzyme-labeling instrument (Infinite F50, TECAN, Switzerland). To ensure reliability, each experiment was conducted in triplicate. The resulting OD values were then compared to a pre-established standard curve to accurately determine the concentrations of the inflammatory markers.

### 2.4 Total RNA and DNA extraction

Liver tissue samples were subjected to total RNA isolation using the Trizol Reagent (Invitrogen Life Technologies, Waltham, MA, United States) following the manufacturer’s protocol. The isolated RNA was stored at −80°C for future transcriptome analysis. For microbiota analysis, total genomic DNA was extracted from intestinal contents using the OMEGA Soil DNA Kit (Omega Bio-Tek, Norcross, GA, United States), adhering to the manufacturer’s instructions. The extracted DNA was preserved at −20°C. The quality and quantity of both RNA and DNA were evaluated using a NanoDrop NC2000 spectrophotometer (Thermo Fisher Scientific, Waltham, MA, United States), and their integrity was confirmed through agarose gel electrophoresis.

### 2.5 Transcriptome and 16S rRNA sequencing

Liver transcriptome sequencing was performed using the GS-FLX+ platform. A cDNA library was constructed from 3 μg of high-quality total RNA, which had been extracted from the liver using the NEBNext Ultra II RNA Library Preparation Kit (New England Biolabs Inc., Ipswich, Massachusetts, United States) according to the manufacturer’s instructions. The cDNA libraries underwent shearing, purification, end-repair, and adapter ligation, with each step specifically optimized for Illumina sequencing. Library fragments were purified using the AMPure XP system (Beckman Coulter, Beverly, CA, United States), focusing primarily on DNA fragments in the 400–500 bp range. Illumina PCR primers were employed to amplify DNA fragments carrying splice molecules at both ends over 15 PCR cycles. The amplified products were then purified again using the AMPure XP system and quantified with the Agilent 2,100 Bioanalyzer (Agilent, Santa Clara, CA, USA). Sequencing was conducted on the NovaSeq 6,000 platform (Illumina, San Diego, CA, USA) by Shanghai Personal Biotechnology Co. Ltd., China, resulting in 125–150 bp paired-end reads.

The V3-V4 hypervariable regions of the bacterial 16S rRNA gene were amplified using the forward primer 338F (5’-ACTCCTACGGGAGGCAGCA-3′) and the reverse primer 806R (5’-GGACTACHVGGGTWTCTAAT-3′) in a thermocycler PCR system (GeneAmp 9,700, ABI, United States). The PCR reactions were conducted in triplicate using a 25 μL reaction mixture that included 1 μL of DNA template extracted from intestinal contents, 5 μL of buffer, 2 μL of dNTPs, 0.25 μL of Fast Pfu DNA Polymerase, 1 μL of each primer, and 14.75 μL of nuclease-free water. The PCR amplification protocol involved an initial denaturation at 98°C for 3 min, followed by 25 cycles of denaturation at 98°C for 30 s, annealing at 53°C for 30 s, and extension at 72°C for 45 s, concluding with a final extension at 72°C for 10 min. The PCR amplicons were purified using Vazyme VAHTSTM DNA Clean Beads (Vazyme, Nanjing, China) and quantified with the Quant-iT PicoGreen dsDNA Assay Kit (Invitrogen, Carlsbad, CA, USA). After individual quantification, the amplicons were pooled in equal amounts, and paired-end sequencing with read lengths of 2 × 250 bp was performed on the Illumina NovaSeq platform using the NovaSeq 6,000 SP Reagent Kit (500 cycles) at Shanghai Personal Biotechnology Co., Ltd., Shanghai, China.

### 2.6 Transcriptome analysis

We utilized fastp (version 0.23.4) to filter out adapter sequences and low-quality reads from the raw data, ensuring high-quality sequences (Chen et al., 2018). The filtered reads were aligned to the goose reference genome (GooseV1.0), obtained from the Ensembl Genome Browser,^1^ using HISAT2 (version 2.1.0) (Kim et al., 2019). Read count values for each gene were initially computed with HTSeq (version 0.9.1) as a measure of expression and then standardized using FPKM (Anders et al., 2014). Differential gene expression analysis was performed with DESeq2 (version 1.38.3) (Wang et al., 2009), defining significance as an absolute |log2FoldChange| > 1 and *p* < 0.05, with *p*-values adjusted using the Benjamini and Hochberg (1995) false discovery rate method. Additionally, bidirectional clustering analysis of all distinct genes across samples was conducted using the R package Pheatmap (version 1.0.12) (Love et al., 2014). GO and Kyoto Encyclopedia of Genes and Genomes (KEGG) pathway enrichment analyses were performed with topGO (version 2.50.0) (Ashburner et al., 2000) and ClusterProfiler (version 4.6.0) (Yu et al., 2012), respectively, applying a significance threshold of *p* < 0.05 for both analyses.

### 2.7 16S rRNA gene bioinformatics analyses

Microbiome bioinformatics analysis was performed using QIIME2 (version 2022.11) in accordance with the official tutorials.^2^ The raw sequence data were demultiplexed, and primers were trimmed using the cutadapt plugin (Martin, 2011). Subsequently, the sequences were quality-filtered, denoised, merged, and chimeras were removed using the DADA2 plugin (Callahan et al., 2016). Alpha-diversity indices, including Chao1, Observed Species, Shannon, and Simpson, were calculated from the ASV table in QIIME2 and visualized using box plots. Beta-diversity, representing genus complexity, was assessed through principal coordinates analysis (PCoA) (Ramette, 2007) and cluster analysis within QIIME2. Linear discriminant analysis effect size (LEfSe) was employed to identify taxa with differential abundance across groups, utilizing default parameters (Segata et al., 2011). Microbial functions were predicted using PICRUSt2 (Douglas et al., 2020), referencing the MetaCyc^3^ and KEGG^4^ databases. Finally, the metagenomeSeq method was applied to identify significant differences in metabolic pathways between groups.

### 2.8 Analysis of the correlations between the gut microbiota and liver DEGs

To investigate the interaction between the gut and liver within the gut-liver axis during fasting and refeeding, we analyzed the correlation between DEGs obtained from transcriptome analysis and the relative abundance of gut microbiota at the species (top 50). Spearman’s correlation analysis was employed across three groups of geese (CON, Fasted, and Refeed) using the mothur software.^5^ The correlation coefficient matrix revealed rho values ranging from −1 to 1. A rho value between −1 and 0 signifies a negative correlation, while values from 0 to 1 represent a positive correlation. A rho of 0 indicates no correlation. Significant correlations |rho| > 0.8 and *p* < 0.01 was constructed and visualized using Cytoscape software (Shannon et al., 2003).

### 2.9 Statistical analysis

Serum biochemical indicators and candidate gene expression data were organized and analyzed using Microsoft Excel 2007. Statistical analyses were performed with SPSS version 26.0. A one-way ANOVA was conducted, followed by Duncan’s multiple comparison test for post-hoc analysis. Statistical significance was defined as *p* < 0.05, with differences considered nonsignificant if *p* > 0.05.

## 3 Results

### 3.1 Effect of fasting and refeeding on circulating immune and inflammatory markers concentrations

**Figure 1:**
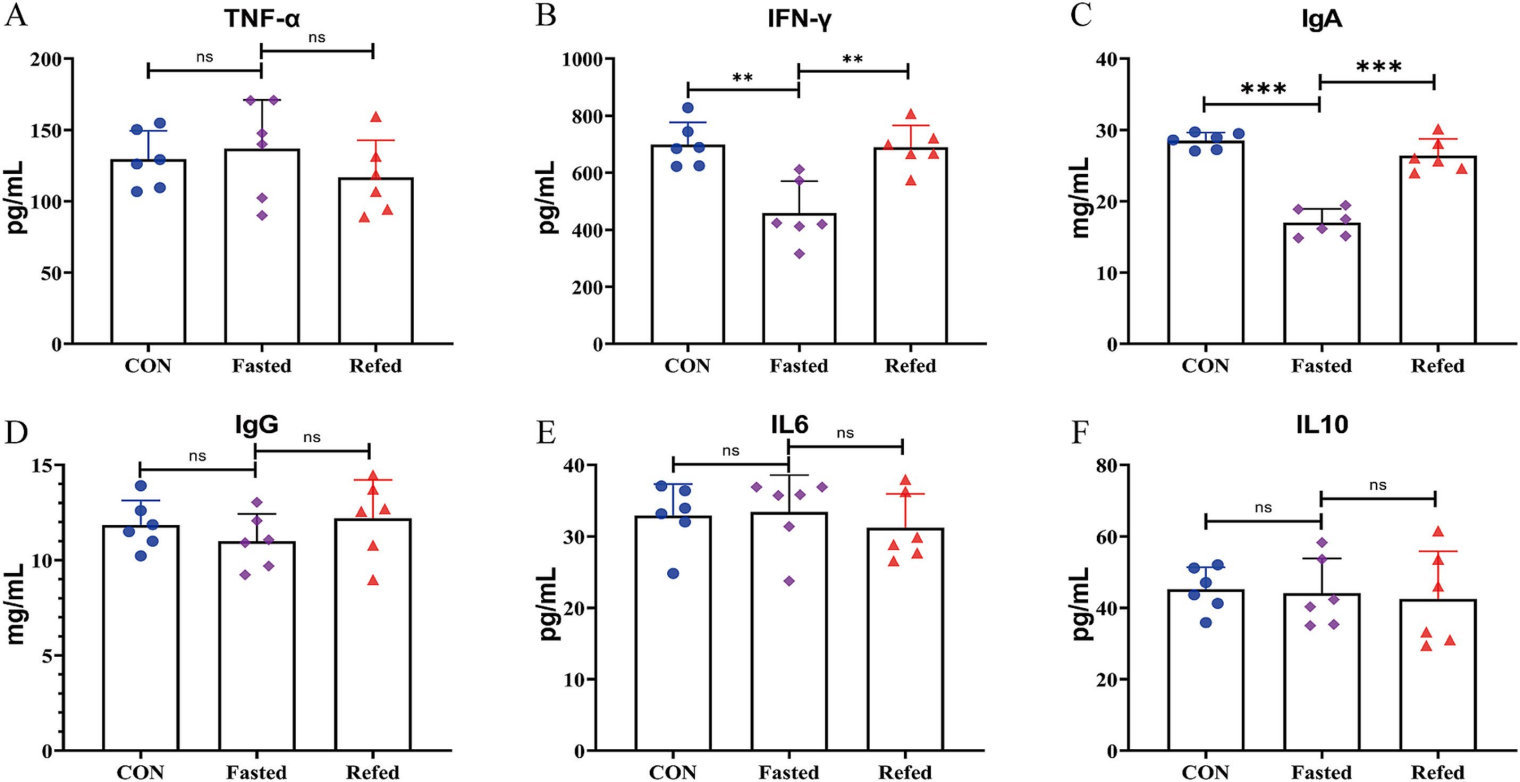
Blood immune and inflammatory markers concentrations of goose under fasting and refeeding. Serum Tumor Necrosis Factor-alpha (TNF-*α*) **(A)**, Interferon-gamma (IFN-*γ*) **(B)**, Immunoglobulin A (IgA) **(C)**, Immunoglobulin G (IgG) **(D)**, Interleukin-6 (IL6) **(E)**, and Interleukin-10 (IL10) **(F)**; CON: Control group; Fasted: 24 h fasting; Refed: 24 h of fasting followed by 3 h of refeeding; *n* = 6; ns > 0.05, **p < 0.01, ***p < 0.001.

Figure 1 presents the concentrations of circulating immune and inflammatory markers (IgA, IgG, TNF-*α*, IFN-*γ*, IL6, and IL10) in geese during both fasting and refeeding periods. IgA levels significantly dropped after 24 h of fasting but returned to baseline within 3 h of refeeding, as shown in Figure 1C. In contrast, IgG levels remained stable throughout both phases, as depicted in Figure 1D. Of the inflammatory markers analyzed, only IFN-γ showed a notable decrease after 24 h of fasting, with levels normalizing within 3 h of refeeding (Figure 1B). No significant changes were observed in the concentrations of TNF-α, IL6, or IL10 during the fasting or refeeding periods (Figures 1A,E,F). These findings indicate that fluctuations in energy and nutrient availability due to fasting and refeeding predominantly affect specific circulating immune and inflammatory markers, particularly IgA and IFN-γ, in geese. This differential response highlights the complex nature of immune and inflammatory regulation in relation to nutritional changes.

### 3.2 Analysis of differential gene expression in goose liver tissue

**Figure 2:**
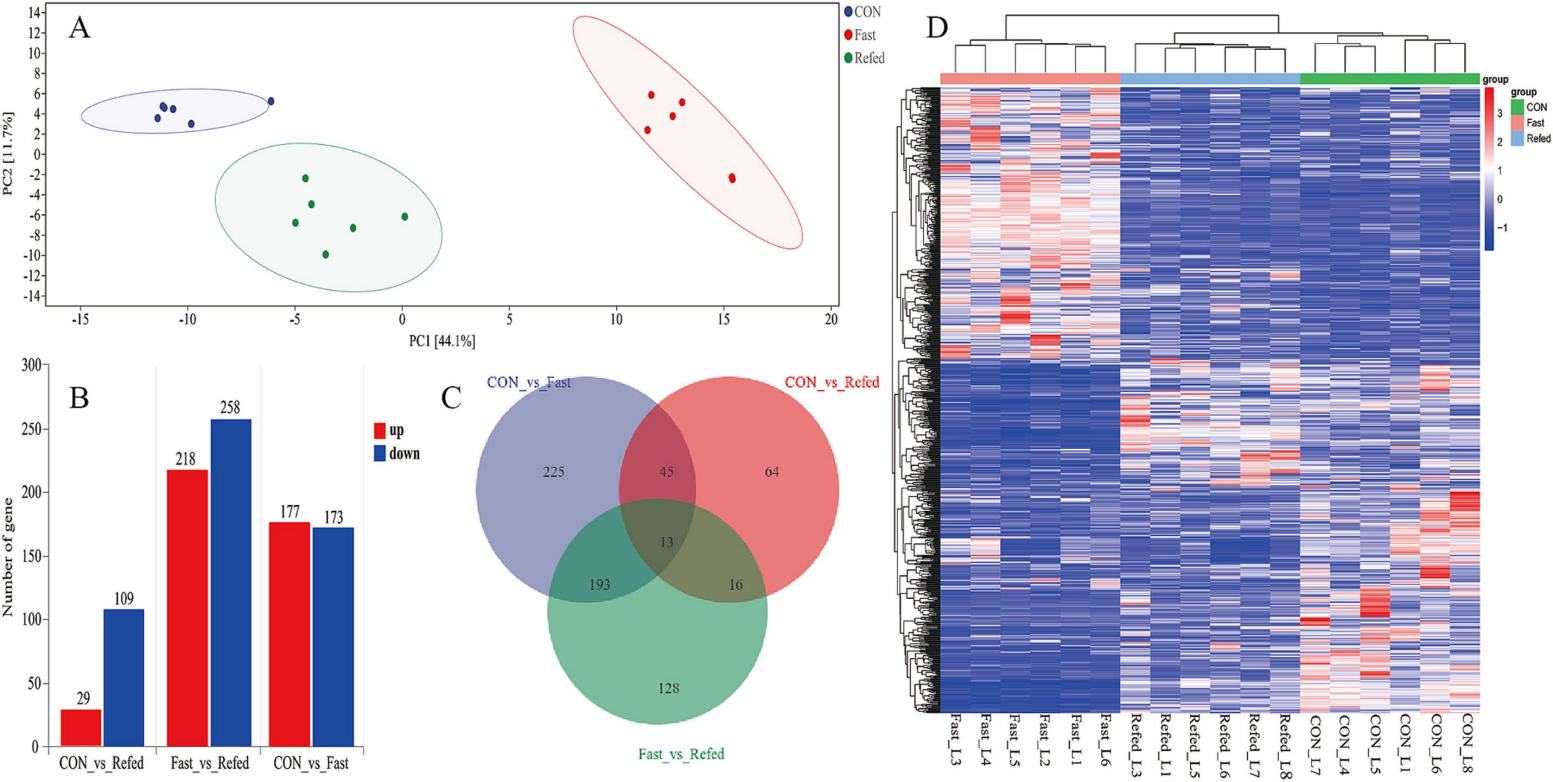
Overview of transcriptome sequencing of the goose livers. Principal component analysis (PCA) conducted for each mRNA-Seq sample **(A)**, Identification of differentially up-regulated and down-regulated genes in each group **(B)**, Venn diagram showing the intersection for the differentially expressed genes between groups **(C)**, Hierarchical clustering analysis of differential gene expression (DGE), with higher expression levels represented by shades of red and lower expression levels depicted in shades of steel blue **(D)**; CON: Control group; Fasted: 24 h fasting; Refed: 24 h of fasting followed by 3 h of refeeding; *n* = 6.

To explore the effects of fasting and refeeding on liver tissue transcription profiles in geese, RNA-Seq analysis was conducted on liver samples from three groups: control (CON), fasting (fasted), and refeeding (refed), with six samples in each group (*N* = 6). Principal component analysis (PCA) revealed distinct separation between the groups, displaying three clear clustering patterns (Figure 2A). Hierarchical clustering of differentially DEGs further supported the PCA results, confirming three clusters among the groups and showing significant clustering consistency among biological replicates within each group (Figure 2D). In the fasted group, 858 DEGs were identified compared to the control, with 431 genes upregulated and 427 downregulated (Figure 2B). Meanwhile, comparison between the refeeding and fasted groups showed 732 DEGs, with 299 genes upregulated and 333 downregulated (Figure 2B). When comparing the refeeding group to the control, 242 DEGs were found, with 66 genes upregulated and 176 downregulated in the refeeding group (Figure 2B). A Venn diagram (Figure 2C) illustrated the overlap of DEGs across the different comparisons. Specifically, 413 DEGs were shared between the CON vs. fasted and fasted vs. refed comparisons, 139 DEGs overlapped between the CON vs. fasted and CON vs. refed comparisons, 56 DEGs were common between the fasted vs. refed and CON vs. refed comparisons, and 37 genes were common across all three comparisons (Figure 2C).

### 3.3 GO and KEGG enrichment analysis for DEGs in goose liver tissue

**Figure 3:**
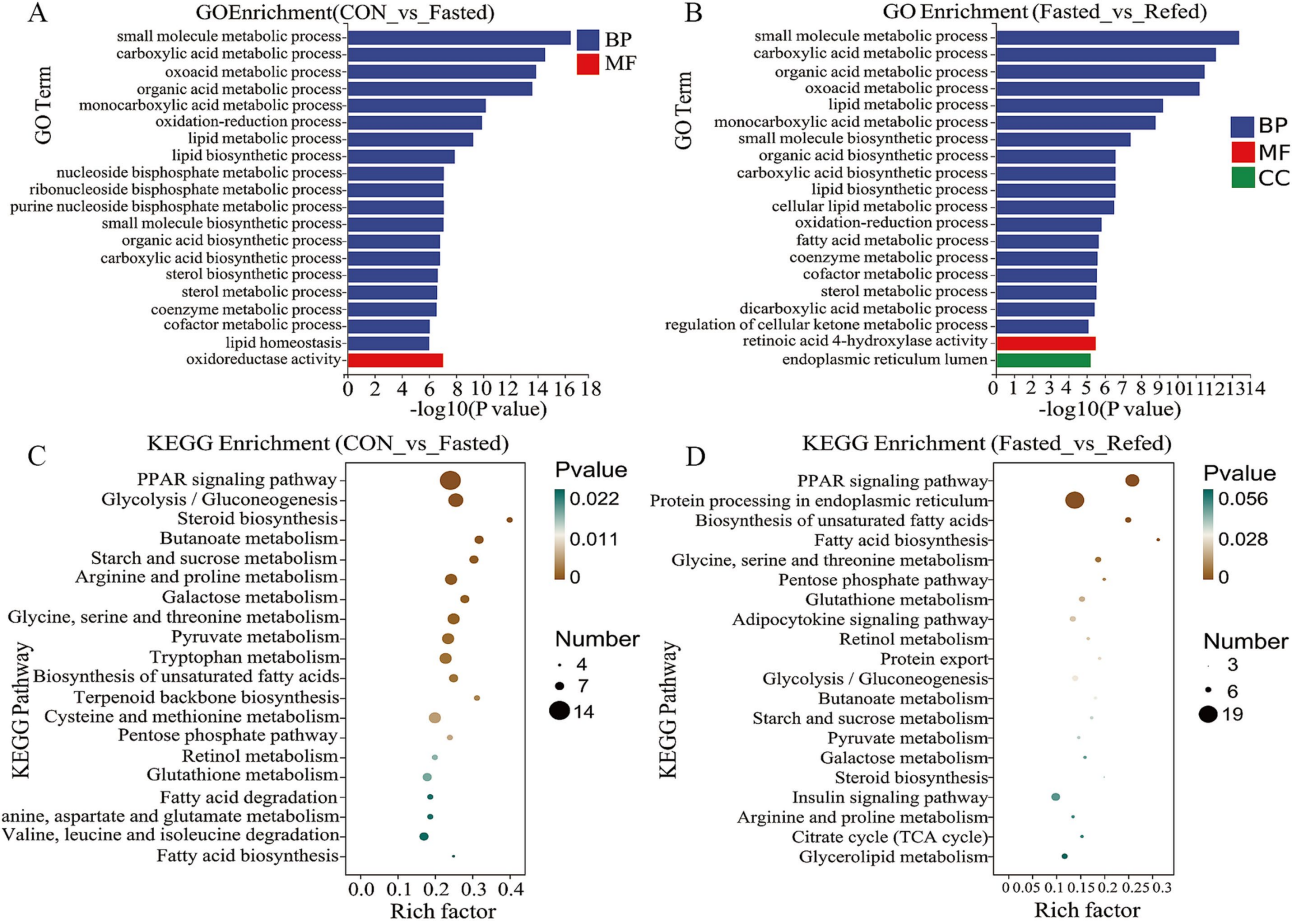
Analysis of GO and KEGG enrichment in DEGs in the liver of goose under fasting and refeeding. The GO annotation terms are divided into three main categories: biological processes (BP), cellular components (CC) and molecular functions (MF). The GO classification map of the liver was generated to compare CON and Fasted **(A)**, as well as Fasted and Refed **(B)**. Top 20 enriched KEGG pathways of the liver was generated to compare CON and Fasted **(C)**, as well as Fasted and Refed **(D)**; CON: Control group; Fasted: 24 h fasting; Refed: 24 h of fasting followed by 3 h of refeeding; *n* = 6.

To evaluate the impact of fasting and refeeding on gene expression in goose liver tissue, we conducted a GO enrichment analysis to explore the roles of differentially DEGs in biological processes (BP), molecular functions (MF), and cellular components (CC) (Figures 3A,B). As illustrated in Figure 3A, the DEGs between the control and fasted groups were mainly associated with small molecule metabolic processes, carboxylic acid metabolism, oxoacid metabolism, and three lipid-related processes: lipid metabolism, lipid biosynthesis, and lipid homeostasis. On the other hand, DEGs between the fasted and refed groups were primarily linked to small molecule metabolic processes, carboxylic acid metabolism, organic acid metabolism, and three lipid-related processes: lipid metabolism, lipid biosynthesis, and fatty acid metabolism (Figure 3B). In addition, we conducted KEGG pathway enrichment analysis on the DEGs identified from the liver tissue transcriptome sequencing. The top 20 enriched KEGG pathways for each comparison are presented in Figures 3C,D. KEGG analysis revealed that the three most significantly enriched pathways in the comparison between the control and fasted groups were the PPAR signaling pathway, Glycolysis/Gluconeogenesis, and Tryptophan metabolism (Figure 3C). When comparing the fasted and refed groups, the most enriched pathways were Protein processing in the endoplasmic reticulum, the PPAR signaling pathway, and the Insulin signaling pathway (Figure 3D).

### 3.4 Identification of key regulatory genes governing fasting and refeeding in the liver

**Figure 4:**
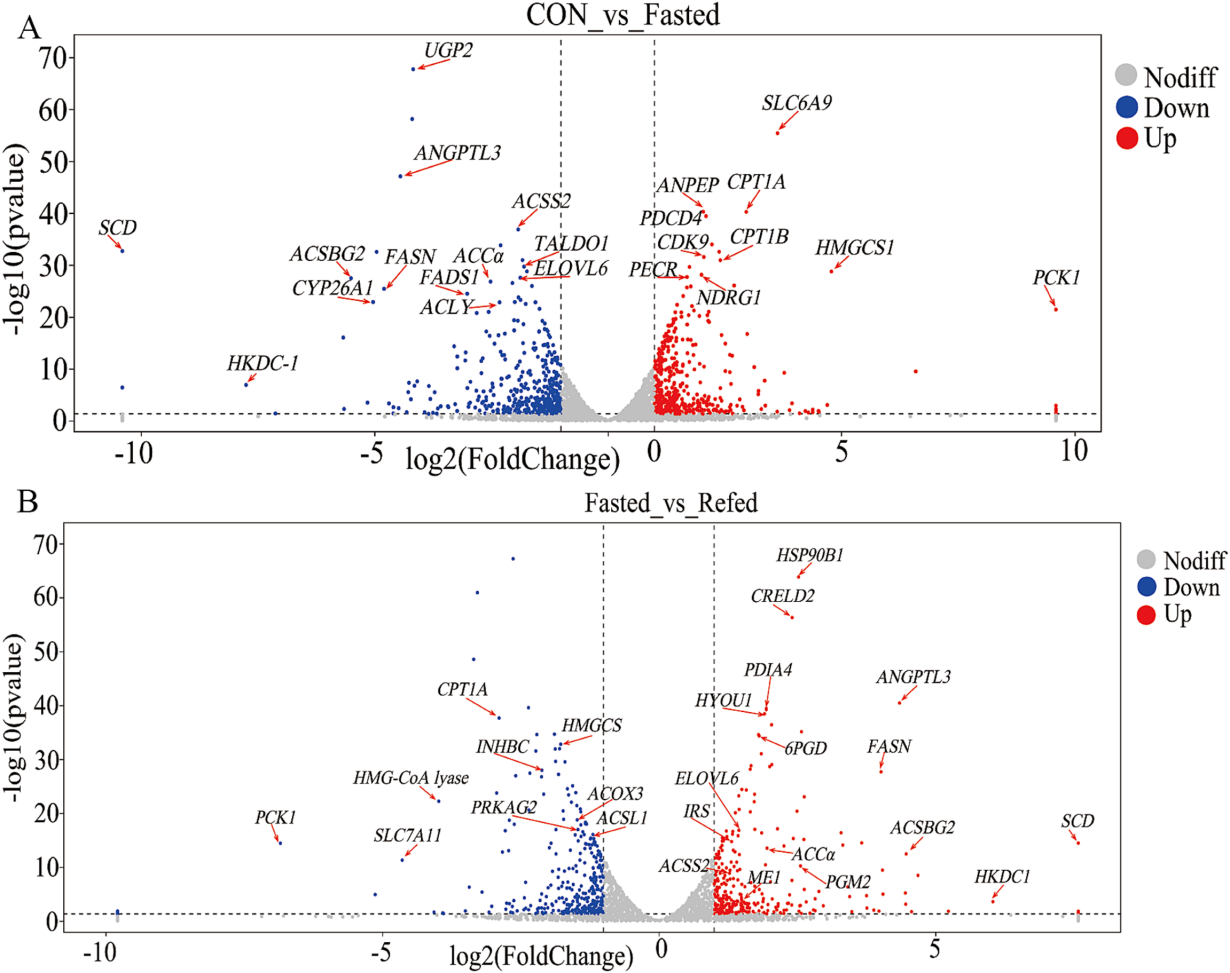
Volcano plot of DEGs in the liver of goose under fasting and refeeding. Upregulated and downregulated DEGs are shown as red and blue dots, respectively. Genes are marked with red arrows and abbreviations. The DEGs of the liver was generated to compare CON and Fasted **(A)**, as well as Fasted and Refed **(B)**; CON: Control group; Fasted: 24 h fasting; Refed: 24 h of fasting followed by 3 h of refeeding; *n* = 6.

Our research aims to clarify the key regulatory genes that govern liver responses to short-term changes in nutrient and energy availability during fasting and refeeding. Through the analysis of a liver RNA-seq database from geese, we identified 23 genes that show differential expression in the livers of fasted geese compared to a control group (Figure 4A, Supplementary Table S1). Among these, 10 genes were upregulated, including solute carrier family 6 member 9 (*SLC6A9*), carnitine palmitoyltransferase 1A (*CPT1A*), alanyl aminopeptidase, membrane (*ANPEP*), programmed cell death 4 (*PDCD4*), 3-hydroxy-3-methylglutaryl-CoA lyase (*HMGCS1*), phosphoenolpyruvate carboxykinase 1 *(PCK1),* carnitine palmitoyltransferase 1B (CPT1B), cyclin-dependent kinase 9 (*CDK9*), peroxisomal trans-2-enoyl-CoA reductase (*PECR*), and N-myc downstream regulated 1 (*NDRG1*). These genes are involved in several key pathways, including PPAR signaling, fatty acid degradation, peroxisome proliferation, and stress responses. On the other hand, 13 genes were downregulated, such as UDP-glucose pyrophosphorylase 2 (*UGP2*), angiopoietin-like 3 (*ANGPTL3*), acyl-CoA synthetase bubblegum family member 2 (ACSBG2), ELOVL fatty acid elongase 6 (*ELOVL6*), stearoyl-CoA desaturase (*SCD*), acyl-CoA synthetase short-chain family member 2 (*ACSS2*), acetyl-CoA carboxylase alpha (ACCα), fatty acid synthase (FASN), fatty acid desaturase 1 (*FADS1*), hexokinase domain containing 1 (*HKDC1*), cytochrome P450 family 26 subfamily A member 1 (*CYP26A1*), transaldolase 1 (*TALDO1*), and ATP citrate lyase (*ACLY*). These genes are primarily involved in processes such as fatty acid biosynthesis, unsaturated fatty acid synthesis, glycolysis/gluconeogenesis, and the insulin signaling pathway. Furthermore, a comparative analysis between the fasting and refeeding groups revealed differential gene expression in the liver, with 27 genes showing significant changes (Figure 4B and Supplementary Table S2). Of these, 18 genes were upregulated, including *HSP90B1* (heat shock protein 90 beta family member 1), *CRELD2* (cysteine-rich with EGF-like domains 2), *ANGPTL3* (angiopoietin-like 3), *FASN* (fatty acid synthase), *PDIA4* (protein disulfide isomerase family A member 4), *HYOU1* (hypoxia up-regulated 1), *ACSBG2* (acyl-CoA synthetase bubblegum family member 2), *FABP7* (fatty acid binding protein 7), *ME1* (malic enzyme 1), *ACCα* (acetyl-CoA carboxylase alpha), *IRS1* (insulin receptor substrate 1), *HKDC1* (hexokinase domain containing 1), *ELOVL6* (ELOVL fatty acid elongase 6), *6PGD* (phosphogluconate dehydrogenase), *ELOVL5* (ELOVL fatty acid elongase 5), *PGM2* (phosphoglucomutase 2), and *ACSS2* (acyl-CoA synthetase short chain family member 2). These upregulated genes are primarily associated with pathways involved in fatty acid biosynthesis, insulin signaling, and related metabolic processes. In contrast, nine genes showed decreased expression, including *CPT1A* (carnitine palmitoyltransferase 1A), *HMGCS1* (3-hydroxy-3-methylglutaryl-CoA synthase 1), *ACOX3* (acyl-CoA oxidase 3, pristanoyl), *ACSL1* (acyl-CoA synthetase long chain family member 1), *PCK1* (phosphoenolpyruvate carboxykinase 1), *PRKAG2* (protein kinase AMP-activated non-catalytic subunit gamma 2), *INHBC* (inhibin subunit beta C), *HMG*-*CoA* lyase (3-hydroxy-3-methylglutaryl-CoA lyase), and *EPHA1* (ephrin A1). These genes are linked to pathways such as *PPAR* signaling, fatty acid degradation, peroxisome proliferation, and glycolysis/gluconeogenesis. Notably, during fasting, genes such as *CPT1A*, *HMGCS1*, and *PCK1* are upregulated, but their expression levels drop during refeeding. Conversely, genes like *ANGPTL3*, *ACSBG2*, *ELOVL6*, *SCD*, *ACSS2*, *ACCα*, and *FASN* are downregulated during fasting but become upregulated during refeeding.

### 3.5 Microbial diversity and composition changes during fasting and refeeding

**Figure 5:**
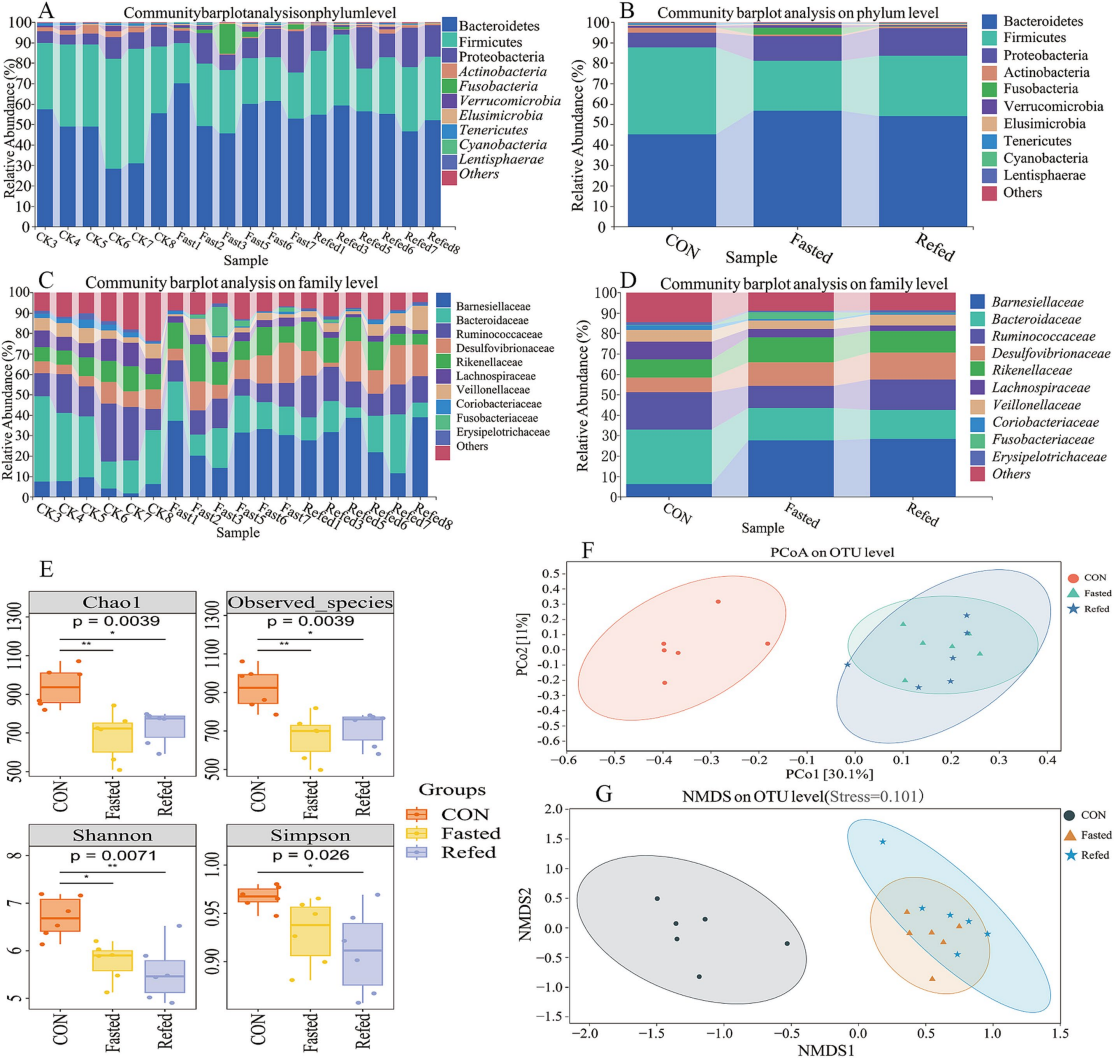
Microbial diversity and composition changes in the rectal microbiota of geese during fasting and refeeding. Community barplot analysis on phylum level across individuals **(A)**, Community barplot analysis on phylum level across groups **(B)**, Community barplot analysis on family level across individuals **(C)**, Community barplot analysis on family level across groups **(D)**, The analysis of alpha diversity **(E)**, Principal coordinates analysis (PCoA) plots of beta-diversity were generated from a total OUTs **(F)**, Nonmetric Multidimensional Scaling (NMDS) plots of beta-diversity were generated from a total OUTs **(G)**; CON, Control group; Fasted, 24 h fasting; Refed, 24 h of fasting followed by 3 h of refeeding; *n* = 6.

This study investigated the effects of fasting and refeeding on the gut microbiota structure in geese using 16S rRNA gene sequencing technology. The rectal segment microbiota was systematically analyzed during the ad libitum feeding, fasting, and refeeding phases. The results indicated that Bacteroidetes, Firmicutes, and Proteobacteria were the dominant phyla in the intestinal microbiomes across all three groups. Notably, fasting geese showed a significant increase in the abundance of Bacteroidetes and Proteobacteria, along with a pronounced decrease in Firmicutes, when compared to the control group. This pattern persisted in the refeeding groups as well (Figures 5A,B). Furthermore, fasting and refeeding phases significantly impacted the family-level microbiota composition, with a notable increase in the relative abundance of Barnesiellaceae, Rikenellaceae, and Desulfovibrionaceae, while the abundance of Bacteroidaceae and Ruminococcaceae decreased compared to the control (CON) group (Figures 5C,D). Alpha-diversity, evaluated using the Chao1 and Observed Species indices for richness, as well as the Shannon and Simpson indices for diversity (Figure 5E), revealed that both richness and diversity were significantly lower in the fasting and refeeding groups compared to the control group (*p* < 0.01 and *p* < 0.05, respectively). These findings suggest that fasting and refeeding considerably decrease microbial richness and diversity in the goose gut. Additionally, beta diversity was assessed using Principal Coordinate Analysis (PCoA) and Nonmetric Multidimensional Scaling (NMDS) to compare the groups (Figures 5F,5G). While the control group formed a distinct cluster, no clear clustering or significant differences were observed in the microbial composition between the fasting and refeeding groups. These results underscore the substantial impact of fasting and refeeding on the diversity and composition of intestinal microorganisms in geese.

### 3.6 Analysis of intestinal microbial species and functional differences during fasting and refeeding

**Figure 6:**
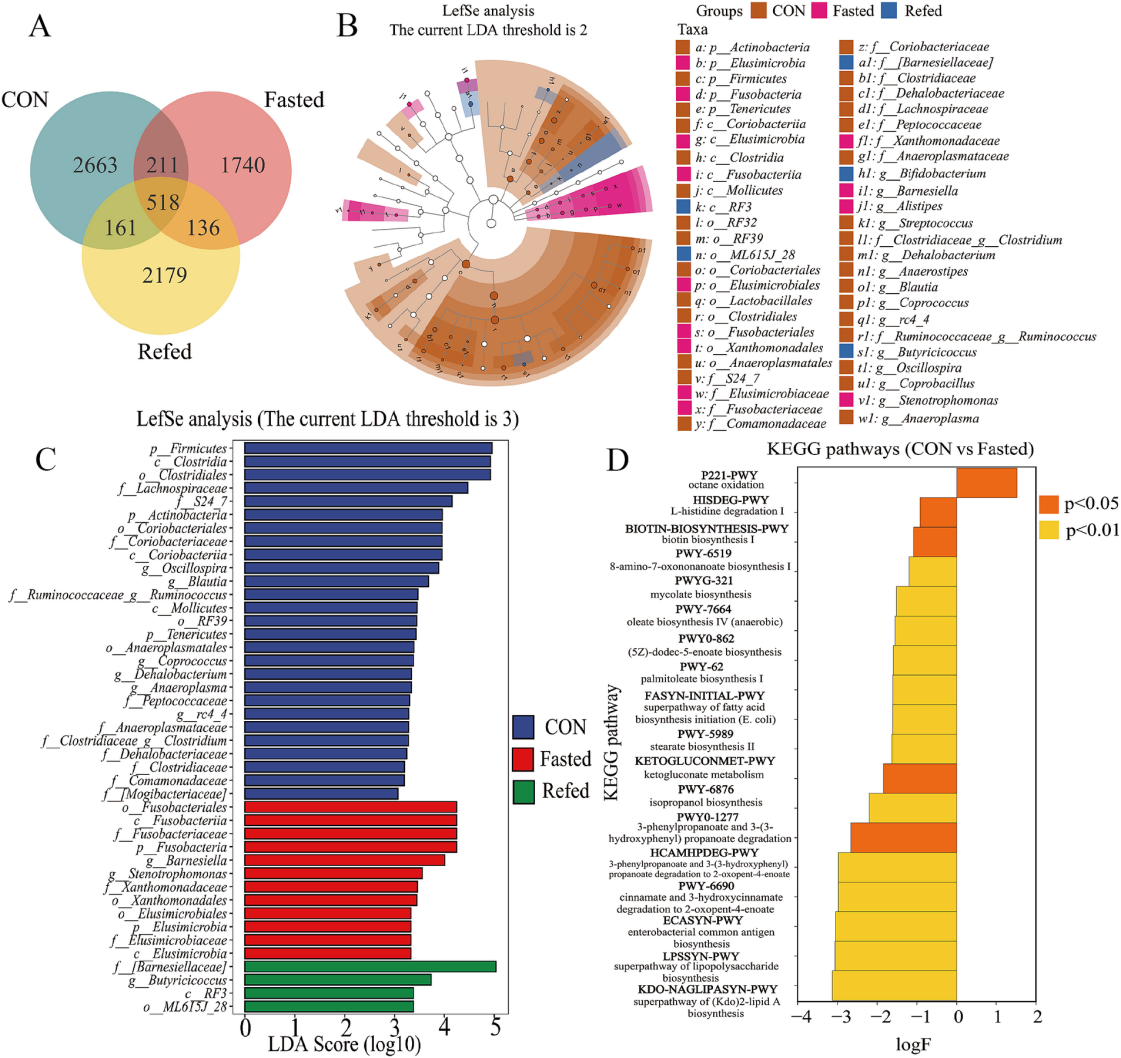
Analysis of intestinal microbial species and functional differences during fasting and refeeding. Venn diagram showing the intersection of OTUs among different groups **(A)**, Taxonomic cladogram generated from LefSe analysis coupled with effect size (LDA > 3) showing difference in microbiota profile during fasting and refeeding **(B)**, Taxonomic abundances generated from LefSe analysis coupled with effect size (LDA > 3) showing difference in microbiota profile during fasting and refeeding **(C)**, The enriched KEGG pathways that changed significantly (*p* < 0.05) between CON and Fasted **(D)**; CON: Control group; Fasted: 24 h fasting; Refed: 24 h of fasting followed by 3 h of refeeding; *n* = 6.

A Venn diagram was subsequently constructed to illustrate the intersections of operational taxonomic units (OTUs) among different groups of geese (Figure 6A). Analysis of this diagram revealed that a total of 518 OTUs were shared among all groups. The control group exhibited 2,663 unique OTUs, while the fasting and refeeding groups displayed 1,740 and 2,179 unique OTUs, respectively (Figure 6A). In summary, these findings suggest that the gut microbiota undergoes rapid changes in response to variations in food availability. Additionally, Linear Discriminant Analysis (LDA) and LEfSe were employed to identify specific bacterial phyla associated with the CON, Fasting, and Refeeding groups, aiming to determine the key taxa driving variations between these groups (see Figures 6B,C). The LEfSe analysis identified a diverse set of 43 distinct species significantly contributing to variations in relative abundance across the three groups. Each species was marked by an LDA score greater than 3 and a *p*-value less than 0.05. Among this complex microbial diversity, the CON group exhibited 27 distinct taxa, while the fasting and refeeding groups contained 12 and 4 unique taxa, respectively (refer to Figure 6C). Notably, Actinobacteria, Firmicutes, and Tenericutes were predominantly identified in the gut microbiota of control geese. In contrast, Elusimicrobia and Fusobacteria were significantly more dominant in the gut microbiota of fasting geese. At the genus level, Oscillospira, Blautia, Ruminococcus, Coprococcus, Anaeroplasma, rc4_4, and Clostridium were predominant in the gut microbiota of control geese. Meanwhile, Barnesiella and Stenotrophomonas were notably more prevalent in the gut microbiota of fasting geese, and Butyricicoccus was prominently observed in the gut of refeeding geese (Figure 6B).

To elucidate the differences between the fasting and refeeding groups in comparison to the control group, and to understand how the gut microbiome regulates host metabolism, a functional predictive analysis of the microbiome was conducted using the KEGG database (Figure 6D). The analysis revealed that the fasting group exhibited a significant upregulation of the octane oxidation pathway relative to the control group. In contrast, other metabolic pathways, including the superpathway of lipopolysaccharide biosynthesis, enterobacterial common antigen biosynthesis, and the superpathway of (Kdo)₂-lipid A biosynthesis, were significantly downregulated. There were no significant differences in metabolic pathways observed between the fasting and refeeding groups.

### 3.7 Analysis of the correlations between the gut microbiota and liver DGEs

**Figure 7:**
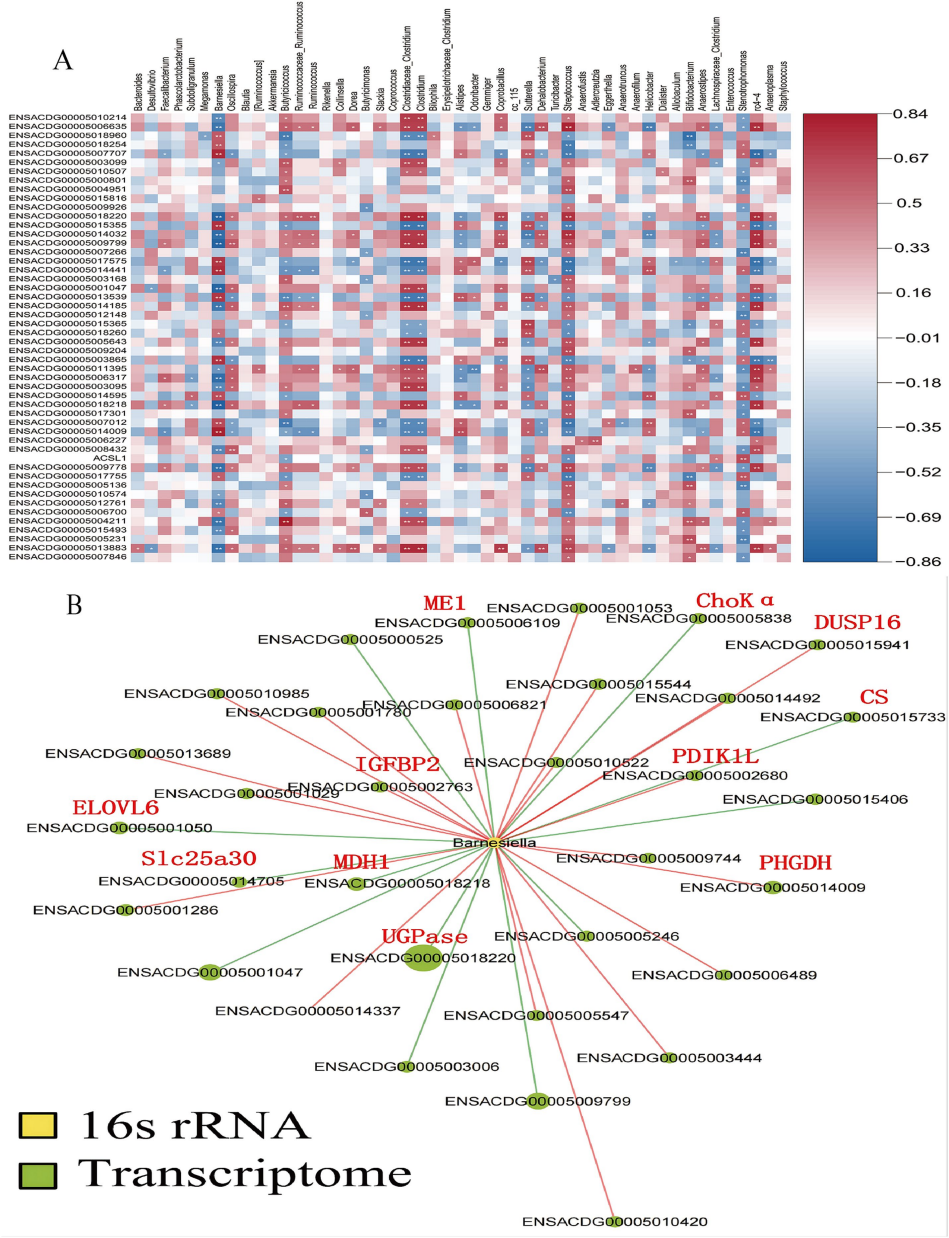
Analysis of the correlations between the gut microbiota and liver DGEs. The correlations between the gut microbiota at the species levels and the liver differentially expressed genes (DEGs) as analyzed by Spearman correlation **(A)**, Changes in the relative abundance of Barnesiella were associated with genes involved in lipid metabolism and glycometabolism. Key genes are marked with red abbreviations **(B)**.

Furthermore, a Spearman’s correlation-based analysis was performed to explore the interactions between the gut microbiota and the liver. Among the 1,161 genes analyzed, genus-gene pairs were identified with a significance level of *p* < 0.05. Figure 7A depicts the correlations between gut microbiota at species levels and the liver’s DEGs as assessed by Spearman correlation. Changes in the relative abundance of Barnesiella were linked with genes involved in lipid metabolism and glycometabolism (Figure 7B). Specifically, genes related to sugar metabolism—such as UDP-glucose pyrophosphorylase (UGPase), malate dehydrogenase (*MDH1*), citrate synthase (*CS*), and malic enzyme 1 (*ME1*)—showed a positive correlation with variations in the relative abundance of Barnesiella (*p* < 0.05). In contrast, genes associated with lipid metabolism, including ELOVL fatty acid elongase 6 (*ELOVL6*), phosphoglycerate dehydrogenase (*PHGDH*), and choline kinase alpha (ChoK*α*), exhibited a negative correlation with changes in the relative abundance of Barnesiella (*p* < 0.05).

## 4 Discussion

Fasting and refeeding are widely used experimental models for studying metabolic regulation and physiological adaptations to variations in nutrient intake. These processes significantly affect various biological systems, including immune functions, liver metabolism, and gut microbiota. This study investigates the complex effects of fasting and refeeding in geese by evaluating immune and inflammatory responses, liver transcriptional changes, and alterations in gut microbiota composition. It provides a thorough analysis of how short-term fluctuations in nutrient availability influence key physiological systems in geese.

### 4.1 Impact of fasting and refeeding on immune and inflammatory markers in geese

The present study investigated the effects of fasting and subsequent refeeding on circulating immune and inflammatory markers in geese, providing novel insights into physiological adaptations to short-term nutritional changes. The research highlights the impact of these states on immune responses, particularly focusing on significant changes in IgA and IFN-*γ* levels, while markers such as IgG, TNF-*α*, IL6, and IL10 showed relative stability. These findings underscore the immune system’s selective responsiveness to fluctuations in energy and nutrient availability and contribute to a broader understanding of how nutritional states influence avian immunity.

A notable finding was the marked reduction in circulating IgA levels after 24 h of fasting, followed by a rapid return to baseline within 3 h of refeeding. IgA is critical for mucosal immunity, and its dynamic response to nutritional changes may reflect a prioritization of immune resource allocation during energy constraints. Similar patterns have been observed in mice, where fasting reduces IgA production in Peyer’s patches due to a migration of naive B cells to the bone marrow, with refeeding restoring IgA levels via the upregulation of CXCL13 by stromal cells (Nagai et al., 2019). In chickens, fasting also reduces sIgA excretion (Parsons et al., 2023). These parallels suggest a conserved mechanism across species in which immunity system is highly sensitive to short-term energy and nutritional shifts. In contrast, circulating IgG levels remained largely unaffected by fasting or refeeding, consistent with findings in mice where systemic immunity, mediated by IgG, demonstrates stability under short-term energy deprivation (Nagai et al., 2019). This stability aligns with the understanding that systemic immunity may be less prone to short-term perturbations but susceptible to chronic or severe energy deficiencies. Among inflammatory markers, only IFN-*γ* exhibited a significant decrease during fasting, followed by a rapid recovery upon refeeding. IFN-γ is pivotal in immune regulation, particularly in macrophage activation and adaptive immunity enhancement. Its decrease during fasting may reflect an adaptive, resource-conserving mechanism where the immune system downregulates pro-inflammatory responses under energy scarcity. Similar reductions in IFN-γ have been reported in fasting humans (Han et al., 2021a, 2021b), supporting the idea that fasting selectively modulates immune responses while conserving resources. The unaltered levels of TNF-α, IL6, and IL10 further support the hypothesis that fasting-induced inflammatory modulation is selective rather than systemic. Studies in other species corroborate that significant changes in these cytokines occur primarily under conditions of substantial energy deficits or weight loss exceeding 5–10% (Mulas et al., 2023; Bastard et al., 2000; Moschen et al., 2010; Liu et al., 2021). These results suggest that short-term fasting primarily impacts components of the immune and inflammatory systems that are metabolically active or directly responsive to changes in energy availability, such as IgA and IFN-γ. The observed resilience of other markers may reflect a threshold effect where more prolonged or severe energy deficits are required to trigger broader inflammatory responses. Additionally, the liver’s role as a metabolic hub and its connection to immune modulation through the gut-liver axis likely play a significant role in these responses, as indicated by previous studies in both poultry and mammals (Hermier, 1997; Wang et al., 2020).

In conclusion, this study highlights the nuanced regulation of immune and inflammatory markers in geese in response to short-term nutritional changes. The selective modulation of IgA and IFN-γ levels underscores the complexity of immune resource allocation during energy fluctuations. Further research should focus on the underlying mechanisms driving these responses and explore the long-term implications of dietary strategies on immune function and health in poultry.

### 4.2 Key regulatory genes and pathways modulating liver response to fasting and refeeding in geese

This study provides a comprehensive analysis of differential gene expression (DGE) in goose liver tissues under fasting and refeeding conditions, revealing transcriptional changes associated with distinct nutritional states. Principal component analysis (PCA) and hierarchical clustering revealed clear separations among the control, fasting, and refeeding groups, each corresponding to distinct transcriptional profiles reflective of different nutritional states. A total of 858 and 732 DEGs were identified between the control and fasted groups and the fasted and refed groups, respectively, with these genes primarily linked to pathways involving energy metabolism, lipid regulation, and stress responses. These findings align with previous studies, which have shown that fasting and refeeding significantly impact transcriptional expression in poultry livers, particularly in pathways related to lipolysis, gluconeogenesis, and lipogenesis (Cogburn et al., 2020; Chen et al., 2021). GO enrichment analysis further revealed that the DEGs between the control and fasted groups were predominantly involved in small molecule metabolism, carboxylic acid metabolism, and lipid-related processes. These findings align with prior research, which suggests that fasting activates metabolic pathways focused on conserving energy and mobilizing stored nutrients (Wang et al., 2015). Similarly, GO terms related to lipid metabolism, fatty acid biosynthesis, and small molecule metabolism were significantly enriched in the comparison between the fasting and refeeding groups, reflecting the liver’s metabolic flexibility in transitioning from catabolism during fasting to anabolism during refeeding (Rui, 2014). Additionally, KEGG pathway enrichment analysis identified significant involvement of the PPAR signaling pathway, known for regulating fatty acid oxidation and lipogenesis in response to metabolic demands (Kersten et al., 2000; Chen et al., 2021). The Glycolysis/Gluconeogenesis pathway was notably enriched during fasting, while the Insulin signaling pathway dominated during refeeding, emphasizing insulin’s crucial role in facilitating glucose uptake and lipid synthesis after refeeding (Saltiel and Kahn, 2001). RNA-seq analysis results revealed that the *CPT1A*, *HMGCS1*, and *PCK1* genes were upregulated during fasting. *CPT1A*, encoding carnitine palmitoyltransferase 1A, is crucial for transporting long-chain fatty acids into the mitochondria for *β*-oxidation, supporting energy production under nutrient-scarce conditions (McGarry and Brown, 1997). HMGCS1, a key enzyme in ketogenesis, plays a vital role in maintaining energy homeostasis during prolonged fasting by generating ketone bodies from fatty acids (Wang et al., 2015). *PCK1*, which catalyzes a rate-limiting step in gluconeogenesis, ensures a steady glucose supply during fasting (She et al., 2000). These gene expression changes illustrate the liver’s adaptation to fasting, shifting from glucose consumption to gluconeogenesis and fatty acid oxidation to meet energy demands. Conversely, genes involved in lipogenesis, such as *ANGPTL3*, *ACSBG2*, *ELOVL6*, *SCD*, *ACSS2*, *ACCα*, and *FASN*, were significantly downregulated during fasting. These genes are associated with lipid biosynthesis, fatty acid elongation, and desaturation (Cogburn et al., 2020; Chen et al., 2021). For example, *ANGPTL3* inhibits lipoprotein lipase, and its downregulation facilitates the release of fatty acids for energy use during fasting (Shimano and Sato, 2017). *ELOVL6* and *SCD*, involved in fatty acid elongation and desaturation, showed reduced expression, indicating a suppression of *de novo* lipogenesis to conserve energy during nutrient deprivation (Matsuzaka and Shimano, 2009). During refeeding, the liver underwent significant transcriptional reprogramming, transitioning from a catabolic state to an anabolic state. Lipogenesis-related genes such as *ANGPTL3*, *ACSBG2*, *ELOVL6*, *SCD*, *ACSS2*, *ACCα*, and *FASN* were upregulated, reversing their fasting-induced downregulation to promote lipid biosynthesis after nutrient restoration. *FASN*, encoding fatty acid synthase, plays a pivotal role in fatty acid synthesis, while *ACCα*, an enzyme that catalyzes the conversion of acetyl-CoA to malonyl-CoA (a critical substrate for fatty acid synthesis), underscores the liver’s metabolic shift toward lipogenesis during refeeding (Brownsey et al., 2006). In contrast, genes involved in fatty acid oxidation and gluconeogenesis, such as *CPT1A*, *HMGCS1*, and *PCK1*, were downregulated during refeeding, reflecting a return to typical metabolic processes prioritizing lipid synthesis over fatty acid oxidation. The reduced expression of *CPT1A* during refeeding decreases mitochondrial fatty acid uptake, favoring energy storage rather than utilization. In summary, this study provides valuable insights into the transcriptional responses of goose liver during fasting and refeeding. The liver’s ability to alternate between catabolic and anabolic states is controlled by a complex network of regulatory genes and pathways, particularly those involved in lipid metabolism, gluconeogenesis, and insulin signaling. These findings enhance our understanding of the metabolic adaptations of avian species to fluctuating nutrient availability and provide a basis for future research into optimizing feeding strategies for improved health and productivity.

### 4.3 Impact of fasting and refeeding on gut microbiota composition and its correlation with liver metabolic genes in geese

This study systematically investigates the effects of fasting and refeeding on gut microbiota diversity and composition in geese, revealing significant changes in microbial communities and their functional profiles. Using 16S rRNA sequencing, we observed a marked reduction in both alpha and beta diversity during the fasting period. Alpha diversity indices, such as Chao1 and Shannon, revealed a significant decline in microbial richness and diversity in both fasting and refeeding groups compared to the control group. These findings align with previous research indicating that nutrient deprivation or caloric restriction reduces microbial diversity, potentially affecting the gut’s resilience and its ability to maintain host health (Cani and Delzenne, 2009; Lozupone et al., 2012). Beta diversity analyses, performed through PCoA and NMDS, further highlighted distinct clustering among the control, fasting, and refeeding groups. However, the lack of significant differences between the fasting and refeeding groups suggests that the refeeding phase may be insufficient to restore the microbiota to its original state, a trend similarly reported in human and animal studies (Sonnenburg and Sonnenburg, 2014).

Fasting-induced shifts in microbial composition included an increase in Bacteroidetes and Proteobacteria and a decrease in Firmicutes, changes consistent with other fasting studies (Sonnenburg and Bäckhed, 2016; Flint et al., 2012). Bacteroidetes, recognized for their ability to degrade complex carbohydrates, increase in abundance under limited nutrient availability, reflecting their metabolic adaptability. Conversely, Firmicutes, associated with enhanced energy extraction from food (Turnbaugh et al., 2006), decline during fasting due to reduced energy intake. The rise in Proteobacteria, often linked to metabolic stress and inflammation (Shin et al., 2015), suggests that fasting may trigger a microbial response aimed at adapting to energy scarcity, potentially influencing host metabolic and immune pathways. An important aspect of this study is the correlation between gut microbiota changes and liver differentially expressed genes (DEGs), emphasizing the gut-liver axis as a critical mediator of host metabolic responses during fasting and refeeding. Notably, the relative abundance of Barnesiella, a genus within Bacteroidetes, was positively associated with genes involved in glycose metabolism, such as *UGPase*, *MDH1*, *CS*, *ME1*. This suggests that Barnesiella may contribute to gluconeogenesis and energy production during fasting by supporting metabolic pathways that compensate for restricted carbohydrate intake (Sonnenburg and Bäckhed, 2016). Conversely, Barnesiella exhibited a negative correlation with genes related to lipid metabolism, incuding *ELOVL6*, *PHGDH*, *ChoKα*, indicating a potential microbial role in suppressing lipid biosynthesis during fasting. This supports the idea that the gut microbiota facilitates a metabolic shift from lipid anabolism to catabolism, prioritizing fat mobilization and oxidation to meet energy demands under nutrient scarcity (Bäckhed et al., 2004). These findings provide valuable insights into how dietary interventions such as fasting influence the gut microbiota and its interplay with host metabolism. The observed reduction in microbial diversity and functional capacity during fasting, coupled with the gut microbiota’s correlations with liver metabolic pathways, underscores the significance of the gut-liver axis in maintaining energy homeostasis. The results highlight how microbial shifts contribute to metabolic adaptations, such as enhanced gluconeogenesis and reduced lipid biosynthesis, during periods of nutritional challenge.

In summary, this study demonstrates that fasting and refeeding profoundly affect gut microbiota composition and diversity, with notable implications for host metabolism. These findings enhance our understanding of gut-liver interactions and provide a foundation for exploring dietary strategies to optimize metabolic health in geese and other species.

## 5 Conclusion

**Figure 8:**
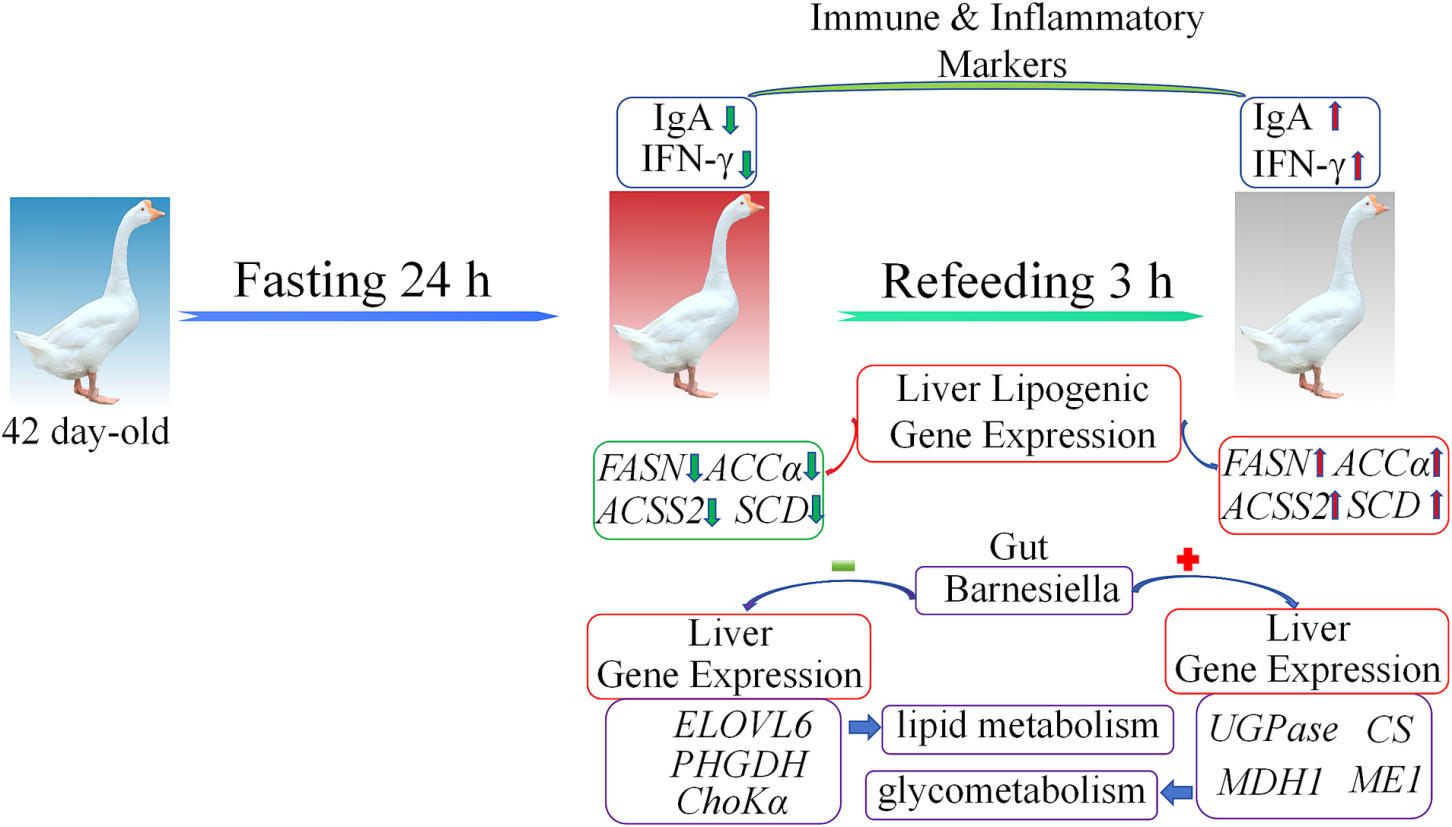
The model summarizing the main findings of this study. Green arrows represent downregulated gene expression, red arrows represent upregulated gene expression, red plus signs indicate positive correlation, and green minus signs indicate negative correlation.

This study demonstrates the profound effects of fasting and refeeding on immune responses, liver gene expression, and gut microbiota composition in geese (Figure 8). Fasting resulted in significant reductions in IgA and IFN-*γ* levels, alongside alterations in gut microbiota diversity, notably an increase in Bacteroidetes and Proteobacteria populations. The liver showed transcriptional shifts, transitioning from gluconeogenesis and fatty acid oxidation during the fasting period to lipogenesis upon refeeding. The observed correlation between gut microbiota and liver metabolic gene expression emphasizes the critical role of the gut-liver axis in maintaining energy homeostasis during nutritional stress. These findings provide valuable insights into how dietary interventions can modulate metabolic health and immune function in poultry.

## Data Availability

The datasets RNA-seq for this study can be found in the NCBI: https://www.ncbi.nlm.nih.gov/bioproject/PRJNA1162240. The datasets 16S rRNA for this study can be found in the NCBI: https://www.ncbi.nlm.nih.gov/bioproject/PRJNA1162434.

## Footnotes

^1^ http://www.ensembl.org/

^2^ https://docs.qiime2.org/2022.11/tutorials/

^3^ https://metacyc.org/

^4^ https://www.kegg.jp/

^5^ http://www.mothur.org
